# RAB5 Hyperactivation Induces APP‐Driven Endolysosomal and Autophagic Dysfunction in Down Syndrome: Reversal by Antisense Oligonucleotides Targeting *App* and *Rab5*

**DOI:** 10.1002/alz70855_102499

**Published:** 2025-12-23

**Authors:** Xu‐Qiao Chen, Xinxin Zuo, Hien T Zhao, William C. Mobley

**Affiliations:** ^1^ University of California San Diego, La Jolla, CA, USA; ^2^ Ionis Pharmaceuticals, Inc., Carlsbad, CA, USA; ^3^ Department of Neurosciences, University of California, San Diego, La Jolla, CA, USA

## Abstract

**Background:**

Down syndrome (DS) significantly increases the risk of Alzheimer's disease (DS‐AD), with dysfunctions in the endolysosomal network (ELN) and autophagy pathways playing central roles in its pathogenesis. Dysregulation of the ELN, particularly involving RAB5 and lysosomal cathepsins, has been implicated in DS‐AD, but the specific role of RAB5 hyperactivation remains poorly understood.

**Methods:**

Postmortem brain samples from individuals with DS, DS‐AD, a partial trisomy 21 case, and the Dp16 DS mouse model were examined to assess the impact of *APP* gene dosage on ELN and autophagy. We measured RAB5 activation, the activity of RAB7 and RAB11, their guanine nucleotide exchange factors (GEFs), lysosomal cathepsins, and autophagy‐related pathways. Additionally, Dp16 mice were treated with *App*‐ and *Rab5*‐specific antisense oligonucleotides (ASOs) to evaluate their therapeutic potential.

**Results:**

Our findings revealed substantial ELN dysfunction in both DS and Dp16 brains, characterized by RAB5 hyperactivation, increased RAB7 and RAB11 activation, elevated levels of their GEFs, and increased lysosomal cathepsin levels—all in an *APP* dose‐dependent manner. Reduced expression of TSC1/2 and hyperphosphorylation of mTOR were associated with impaired autophagy. These abnormalities were absent in a partial trisomy 21 individual with two copies of *APP*. Treatment with ASOs in Dp16 mice restored RAB5 activity, normalized ELN function, and improved autophagic flux, alleviating DS‐AD‐related pathologies including tau hyperphosphorylation, neurotrophin signaling deficits, and synaptic protein loss.

**Conclusion:**

Our results demonstrate that *APP* dose‐driven RAB5 hyperactivation disrupts endosomal Rab cascades, endosome maturation, and autophagy function in DS. Targeting either *APP* or *Rab5* may offer promising therapeutic strategies to restore cellular function and mitigate DS‐AD pathologies.

